# The MPI Emotional Body Expressions Database for Narrative Scenarios

**DOI:** 10.1371/journal.pone.0113647

**Published:** 2014-12-02

**Authors:** Ekaterina Volkova, Stephan de la Rosa, Heinrich H. Bülthoff, Betty Mohler

**Affiliations:** 1 Department of Human Perception, Cognition and Action, Max Planck Institute for Biological Cybernetics, Tübingen, Germany; 2 Graduate School of Neural & Behavioural Sciences, Tübingen, Germany; 3 Department of Brain and Cognitive Engineering, Korea University, Seoul, South Korea; Durham University, United Kingdom

## Abstract

Emotion expression in human-human interaction takes place via various types of information, including body motion. Research on the perceptual-cognitive mechanisms underlying the processing of natural emotional body language can benefit greatly from datasets of natural emotional body expressions that facilitate stimulus manipulation and analysis. The existing databases have so far focused on few emotion categories which display predominantly prototypical, exaggerated emotion expressions. Moreover, many of these databases consist of video recordings which limit the ability to manipulate and analyse the physical properties of these stimuli. We present a new database consisting of a large set (over 1400) of natural emotional body expressions typical of monologues. To achieve close-to-natural emotional body expressions, amateur actors were narrating coherent stories while their body movements were recorded with motion capture technology. The resulting 3-dimensional motion data recorded at a high frame rate (120 frames per second) provides fine-grained information about body movements and allows the manipulation of movement on a body joint basis. For each expression it gives the positions and orientations in space of 23 body joints for every frame. We report the results of physical motion properties analysis and of an emotion categorisation study. The reactions of observers from the emotion categorisation study are included in the database. Moreover, we recorded the intended emotion expression for each motion sequence from the actor to allow for investigations regarding the link between intended and perceived emotions. The motion sequences along with the accompanying information are made available in a searchable MPI Emotional Body Expression Database. We hope that this database will enable researchers to study expression and perception of naturally occurring emotional body expressions in greater depth.

## Introduction

Emotions shape human communication and have been a long-standing subject of research in many fields of science including anthropology, psychology, and neuroscience [1]–[3]. Previous research has largely focused on the examination of perceptual and cognitive processes underlying the recognition of facial emotional expressions. For several decades, body movements have been largely neglected in emotion research although they make an important contribution to emotion recognition and even modulate the interpretation of facial emotion [3], [4]. Recently, however, the body has received more of the deserved attention as an important affective modality [5], [6].

To study body emotion recognition from dynamic stimuli, datasets that allow control over the quality of body movements and the visual representation of data are an important requirement. Over the course of the last few decades multiple datasets have been accumulated. Often collected as part of research reflecting on various aspects of affect in humans, some datasets trace as much as 40 years back [7]–[10]. Due to the progress in recording technology and data storage, most of these corpora and databases have been collected during the last ten years [10]–[31].

Many high quality datasets of human body motion are now available, but we will focus on those that deal with emotional body language. From the perspective of data format, the databases can be primarily collections of motion capture data or video recordings. One of the best known motion capture datasets is from the CMU Graphics Lab (see [11] for one of the initial reports or access the database at http://mocap.cs.cmu.edu). It contains over 2600 full body motion sequences available in different formats. However, a very small proportion of motion sequences in the CMU database has explicit emotional content. The KUG database has been developed by Hwang and colleagues [18] and presents a systematic and well controlled motion capture dataset of non-emotional gestures and simple actions from many actors. The database encompasses gestures common for everyday life, abnormal gestures which are typical for emergency situations, such as falling, and command gestures often used in communication. Ma, Paterson and Pollick [19] have collected an extensive database from 30 actors, where 4080 motion capture sequences encompass waving and other non-verbal actions displayed four emotion categories — *neutral, angry, happy, sad*. This database is a valuable resource, but the motion patterns were captured in purely non-verbal scenarios, thus excluding the context of emotion expression during speech production.

The USC CreativeIT database [26], [32] consists of short, scripted actions performed by pairs of actors. The interactions were recorded with full-body motion capture technology, video and audio. The display of actions was kept as natural and intuitive as possible and the setup itself did not influence the actors' expressive behaviour. Actors did not receive specific instructions as to which emotions to express, neither did they write an acting script for themselves. The database also provides observers' annotations for each recording with regards to the emotion dimensions (valence, activation, and dominance) and performance properties (interest, naturalness, creativity). Body motion of the actors is also analyses for activation and dominance-related information, such as orientation and approach-avoidance behaviours, body and hand movements. Specific emotion expression labels are not provided.

Several new databases and datasets have been developed recently, reflecting on various aspects of emotion expression, such as non-acted emotions expressed while playing video games [33], [34], laughter [35], [36] and pain [37]. In [33], postures of players were collected during a video game session. Importantly, the participants were not aware that their emotional body expressions where the primary point of interest of the study. The captured poses were categorised by naive observers along four emotion categories (*concentrating, defeated, frustrated, and triumphant*) and rated on valence and arousal scales. The physical and perceived properties of the poses were used to build an automatic emotion recognition system, which makes the fact that the poses came from non-acted emotion expression all the more important for ecological validity and accuracy in real-life gaming scenarios.

The Multimodal Multiperson Corpus of Laughter in Interaction (MMLI) [35] is an outstanding resource where the authors combined 3D body position information, facial tracking, audio and video channels as well as physiological data, e.g., respiration sensor. All channels were synchronised, segmented, and annotated to discriminate between laughter and non-laughter. In another study on laughter the authors use motion capture to detect different types of laughter: *hilarious, social, awkward, and fake* [36].

Several studies have created video recording datasets, sometimes combined with audio recordings, to capture emotion expressions typical of communication or free expressions of emotions. Not all of these datasets are available as databases but they make an important contribution to the best practices of data acquisition for emotional body expressions. The aim of most of these studies is to gain a deeper understanding of what properties of motion facilitate attribution of specific emotion categories or general affect dimensions [38], [39]. An important advancement in the understanding of how body motion and body shape influence emotion recognition was made by Atkinson and colleagues [40]–[42]. The stimuli for the perception experiments were originally recorded on video. A well-controlled modification of the video recordings allowed the authors to investigate the perception of emotion from still poses, dynamic stimuli, full-light and point-light displays.

A few other studies have used video recording or motion capture for further feature extraction and automatic emotion recognition from dynamic biological motion or static poses [43] and to study cross-cultural differences in emotion perception [25]. Among the video-based datasets made available for researchers are the GEneva Multimodal Emotion Portrayals (GEMEP) corpus [15] and the Interactive Emotional Dyadic Motion Capture (IEMOCAP) database [22]. GEMEP uses a wide range of emotion categories and a refined emotion induction technique involving pseudo-linguistic sentences. The resulting corpus of 1260 video sequences contains acoustic information, facial expressions and body motion. IEMOCAP is a database of video-recorded and motion captured scripted and spontaneous interactions between dyads of actors. Only facial expressions and general hand movements of one actor in each dyad were motion captured.

Databases of video recordings are restricted to the recorded 2-dimensional data. Additionally, video recordings make the examination of the separate contributions of shape (e.g. height), texture (e.g. colour of clothes and skin), and motion cues to the recognition of body expressions difficult. Even with several cameras positioned at different view points, the retrieval of 3-dimensional body motion is a complicated, noise-prone procedure. State-of-the-art motion capture systems that provide data for body motion in 3-dimensional space and at high frame rates on the other hand allow: (1) extraction of motion trajectories for further analysis and (2) systematic alterations of the position and movement of individual body joints as potential post-processing measures, e.g., for stimuli set generation. Shape and texture cues can be manipulated during the animation process using standard software packages.

Context is another important factor for emotion production and recognition [4], [44]. Not only do context-set emotion expressions allow future research to gain deeper insight into the way people normally express and perceive emotions, but these could also constitute a valuable source of raw data for human motion modelling, e.g., for virtual character or robot animation. In this regard, Oertel et al. [45] have used an intricate setup to record naturalistic conversation in a domestic setting. Having given their participants no restriction over the conversation and interaction flow, the authors have non-intrusively gathered social interaction data by means of audio and video recording and motion capture, creating an ideal corpus for studying naturalistic conversational interaction. A exemplary emotion data collection environment is one where the actors work with a rich set of emotion categories, their emotion expression is induced without strong conscious effort on their part and without unnecessary exaggeration of the expressions. The motion capture setup should not restrict their motion or influence their emotion expression in any way. While purely non-verbal emotion expression has its valid place in human lives, most emotion instances occur during communication and are accompanied by speech production. Thus, for the reasons of ecological validity, it is advantageous to capture motion that occurs during emotional narration. However, the naturalness of emotion expression should ideally be combined with considerable control over the data stream by the experimenter. For example, it is useful for actors and experimenters to agree upon an acting script before the motion capture session.

Using state-of-the-art motion capture technology [46], we have initially recorded a total of 5.4 hours of motion captured narrations performed by amateur actors, from which a total of 86 minutes, split into 1447 motion sequences were selected and included in the new database. In contrast to many previous studies, we mostly captured narrations of whole stories as opposed to separate sentences or actions taken out of context and performed in a randomised order. Special effort has been made to collect motion patterns characteristic of free emotion expression in real life. We used no specific emotion induction technique apart from letting the actors be immersed in the story. The actors were to recount the emotions of the narrator and the characters of the story, imagining they are telling the story to a child or several children. The actors annotated the texts for emotions prior to the motion capture, effectively creating personalised acting scripts.

Most related research has used fewer emotion categories, e.g., the basic emotions [47]. We argue that research on human emotional experience does not have to be constrained to the six basic, universally recognised emotions. We thus developed a list of eleven emotions that were operated with all stages of our research. The list was built by analysing emotion labels from previous work that used more categories than only the basic emotions [15], [30], [38], [39], [48]–[50]. The following criteria were taken into account during the final emotion category list compilation: 1) broader span than the basic emotions, 2) manageable size, 3) balance between negative and positive emotions, and 4) categories frequently used in related research. Our final list of emotion categories used five positive (*amusement, joy, pride, relief, surprise*) and five negative (*anger, disgust, fear, sadness, shame*) emotion categories as well as the *neutral* category.

During the motion capture sessions, the text of the story, the emotion category the actor intended to express at every phrase, and the motion capture data were automatically synchronised during the narration process. The performance was also recorded on video and audio, leaving our actors unaware that only their body motion was of primary interest to the researchers and thus they did not exaggerate the emotion expression through this particular channel. The fact that the motion capture took place in the context of narration, the resulting connections between the original text, its emotion annotation by the actor, their motion patterns and the perception thereof can be valuable for linguists as well. It is also important that our database format is not video but motion capture, which allows to easily process the data, perform qualitative and quantitative analysis, change the presentation display method by altering the underlying body proportions and appearance, modify various motion properties of the whole body or some of its parts in terms of timing and positioning, and have full control of the motion stimuli.

Prior to the creation of the database we conducted an extensive emotion categorisation study using the collected motion sequences. We used upper human body display to present the motion sequences to multiple observers and let them categorise the stimuli using the same set of emotion categories as had been used by the actors. While a detailed account of the results of the categorisation study can be found in [51], a few aspects that specifically fit the scope of this article are reported here for the first time. Importantly, all observers' responses from the categorisation study are included in the database, allowing for comparison between actors' intended emotions and the perceived emotion categories and making it a valuable feature for the users of the database.

Table 1 gives an overview of several databases that include emotional body expressions, allowing for easy comparison with our database. While recent and very detailed reviews [5], [6] provide rich and extensive information about multiple available resources, the databases selected for Table 1 share one or more features with our new database: focus on body motion, motion capture format, large set of emotion categories, naturalness of the produced motion, large number of motion samples. We highlight such properties as the data format, the emotions expressed, the recorded modalities (such as body, facial expressions, speech), the mode in which the emotions were recorded (elicited or natural), the number of the recordings available, the number of actors involved in the data collection, the number of observers (in cases when the recordings were validated with the help of raters), and the context in which the recordings took place. Table 2 lists the emotion categories used in the databases included in Table 1.

**Table 1:** Overview of several databases described in the introduction section.

Name	Format	Emotions or actions	Modalities	Mode	Samples, Actors, Raters	Contexts
CMU MoBo [11]	**M**	actions and interactions	**B**	E	**2605**, 144, 0	various
FABO [17]	V	**10 emotions**	F, **B**	E	**1900**, 23, 0	emotion elicitation by situation vignettes, free expression
KUG [18]	**M**, V	55 actions and gestures	**B**	E	**1080**, 40, 0	directed motion capture in studio
[19]	**M**	actions modulated by 4 emotions	**B**	E	**4080**, 30, 0	emotion and action elicitation by situation vignettes
GEMEP [15], [30]	A, V	**17 emotions**	**B**, F, S	E	**1260**, 10, 57	pseudospeech sentences and a nonverbal vocalization
IEMOCAP [22]	A, **M**, V	6 emotions and 3 affect dimensions	F, **B** (hands)	E, **N**	**10039**, 10, 6	scripted and spontaneous dyadic interactions
USC CreativeIT [26], [32]	A, **M**, V	affect dimensions, interaction tendencies, and performance ratings	**B**, S	E	**>1200**, 19, 5	two-sentence exercise and paraphrases
AffectME [33]	**M**	4 emotions and 2 affect dimensions	**B**	**N**	103, 11, 8	emotions were triggered naturally while playing a video game
[34]	**M**	4 emotions	**B**	**N**	161, 9, 7	Wii Grand Slam Tennis game
[29]	A, V	**8 emotions**	**B**, F, S	**N**	1400, 256, self-report	laboratory-based emotion induction tasks
MMLI [35]	A,**M**, V	laughter episodes	**B**, F, R, S	E, **N**	**500**, 16, 0	word games, humorous videos, tongue twisters
UCL-ILHAIRE [36]	**M**	4 laugher types + non-laughter	**B**	E, **N**	126, 9, 32	word and collaborative games, humorous videos
**Our database**	**M**	**11 emotions**	**B**	E, **N**	**1447**, 8, 55	short scenarios, sentences and narrations

The databases included in this table share one or more features with our new database such as: focus on body motion, motion capture format, rich set of emotion categories (more than the six basic emotions), naturalness of the produced motion, large number of motion samples. These shared features are highlighted in bold. The table highlights the following properties: the major formats the data was recorded in (**A**udio, **M**otion capture, **V**ideo), the emotions expressed, the modalities recorded (**B**ody, **F**ace, **R**espiration, **S**peech), the mode of the emotions (**E**licited vs. **N**aturalistic), the size of the database/dataset, the number of actors, the number of observers/raters, and the context in which the data collection took place. The highlighted initial letters in bold correspond to the code letters in the format, modalities and mode columns. The last row describes the new database for the easiness of comparison. Table 2 lists emotion categories used in the databases.

**Table 2:** Emotion categories used in the databases included in Table 1.

Name	N of categories	Emotion categories
FABO [17]	10 emotions	anger, anxiety, boredom, disgust, fear, happiness, neutral, sadness, surprise, uncertainty
[19]	4 emotions	neutral, angry, happy, sad
GEMEP [15], [30]	17 emotions	amusement, anxiety, cold anger (irritation), despair, hot anger (rage), fear (panic), interest, joy (elation), pleasure (sensory), pride, relief, sadness, admiration, contempt, disgust, surprise, tenderness
IEMOCAP [22]	6 emotions and 3 affect dimensions	neutral, anger, happiness, sadness, frustration, excited; valence, activation, dominance
USC CreativeIT [26], [32]	affect dimensions, interaction tendencies, and performance ratings	valence, activation, dominance; approach-avoidance; interest, naturalness, creativity
AffectME [33]	4 emotions and 2 affect dimensions	concentrating, defeated, frustrated, triumphant; valence, arousal
[34]	4 emotions	happiness, concentrated, high-intensity negative emotion, low-intensity negative emotion
[29]	8 emotions	disgust, surprise, fear, relaxed, sadness, anger, amusement, frustration
MMLI [35]	laughter episodes	laughter, non-laughter
UCL-ILHAIRE [36]	4 laugher types + non-laughter	hilarious laughter, social laughter, awkward laughter, fake laughter, non-laughter
**Our database**	11 emotions	amusement, joy, pride, relief, surprise, anger, disgust, fear, sadness, shame, neutral

The CMU MoBo [11] and the KUG [18] databases were not included in this table because their design was not aimed at capture of emotional body expressions but rather actions and neutral gestures.

In the following sections we present detailed information about the acquired motion data and its properties across individual actors and acting tasks. We also include the results of the emotion categorisation experiment, where each motion sequence was evaluated by multiple observers. The richness of available information — the extended set of emotion categories, the intended and perceived emotion labels provided for each motion sequence, the corresponding text and physical properties — is likely to prove useful for researchers in the fields of motion modelling, human emotion perception, and linguistics.

## Materials and Methods

### Ethics Statement

**Figure 1:**
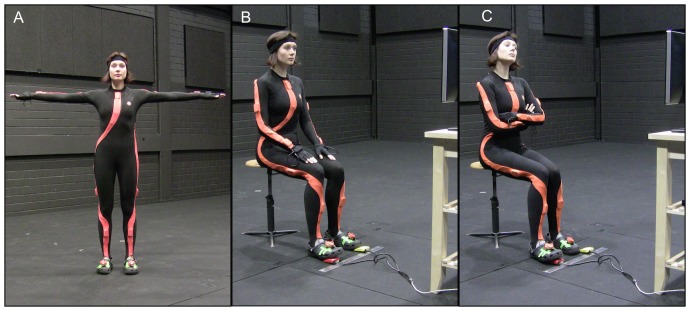
Setup for motion capture sessions. The individual in this figure has given written informed consent (as outlined in PLOS consent form) to publish the photo with the face unmasked. (A) An actor in motion capture Moven Xsens suit, t-pose (B) Acting setup: an actor in neutral pose, stool, pedals and display. (C) An actor expressing *pride*.

The database and the emotion categorisation experiment described later in this manuscript use human volunteers. Informed written consent was obtained prior to any experiment or recording from all participants and actors. Participants and data from participants were treated according to the Declaration of Helsinki. The recording methods of the database and the subsequent validation experiment were approved by the local ethics committee of the University of Tübingen. The individual in this manuscript (Figure 1) has given written informed consent (as outlined in PLOS consent form) to publish their case details.

### Actor Recruitment

The motion capture project was advertised via the local experiment participant network. During the initial introduction to the motion capture scene, each potential actor was asked about their present and past acting experience. Our goal was to record emotional body expressions in an environment as close to natural narration as possible. Since narration is often performed by people in daily life, the exact amount of acting experience was not of primary importance. The major selection criteria were the following: 1) the actor's proficiency in English was to be sufficiently high in order to read English texts fluently and understand the semantic and emotional content of the stories; 2) the actor was to be able to employ all affective media during the narration, such as facial expressions, emotional prosody and body motion.

Three out of eleven initially recruited actors (one male and two female) were excluded from the motion capture sessions at the initial stages. One actor was excluded due to their experienced difficulties in reading the text in a fluent fashion. Two other actors were excluded because they found it very difficult to produce body motion during the narration. This difficulty might have been caused by their previous extensive participation in radio broadcasted programs and similar activities where body motion is not required during emotion expression.

All of the eight remaining actors were amateur actors. The exact and objective estimation of our actors' previous experience was difficult to obtain. The actors differed in acting background (student drama clubs vs. live action role-playing games) and acting activity levels at the time when motion capture sessions took place. We were however careful to avoid recruiting professional theatre actors with many years of experience as they are prone to exaggerate their emotion displays and to use stereotypical motion patterns [52]. Various issues of acting *vs.* emotion elicitation, as well as lay *vs.* professional acting are discussed in detail by Bänziger and colleagues [20], [30] (also see [27]).

### Texts

Most emotion induction techniques have used short situation descriptions or vignettes to aid enactment of various emotions during the recording sessions. One drawback of this approach is that actors often have to switch between emotions randomly. In our study we asked our actors to perform narrations of original fairy tale stories. Each recruited actor was asked to choose three texts out of nine pre-selected fairy tales. The texts of the stories were taken from a large collection of fairy tales recorded by Andrew Lang in a collection of twelve books, published between 1889 and 1910. All nine stories were thus written in unabridged English in consistent style and Andrew Lang's language, eloquent and poetic, was likely to trigger various emotions. The actors familiarised themselves with the texts and then read them out loud.

The actors' speech was recorded for further processing. At this point it was important that the actor read the texts at a speed appropriate for narration. The texts were first split into sentences, then each sentence was split into utterances according to the acoustic pauses in actors' recorded speech. The duration of the pauses used for text splitting was set between 150 ms and 250 ms as this duration usually corresponds to brief pauses between clauses but is too long for inter-lexical pauses. For more detail on prosodic pauses duration see [53]–[55].

The resulting split narrations, 24 in number, were on average almost three hundred utterances long (M = 298.5, SD = 36.09), each utterance containing a few word tokens (M = 6.84, SD = 2.59). The split narrations were then provided as input to our custom-made online annotation tool (www.epetals.org). The actors were asked to use the annotation system and assign one of the eleven emotion labels to each utterance, thus effectively creating personalised acting scripts for themselves. The order of the utterances during the annotation process corresponded to the natural flow of the text. The annotations typically employed the full range of available emotion categories for each annotation (a minimum of eight categories occurred in only one of the annotations). The frequency of categories varied greatly, *neutral* naturally being the most frequent emotion and *shame* the least frequent. These acting scripts were then presented to the actors during the motion capture sessions with the help of PsychoPy software [56].

During all stages of the motion data collection process, starting with the audio recordings, the actors were asked to imagine that they were narrating the stories to a child or children, which placed them into a socialisation scenario, where they could reflect on which emotions should be expressed at any moment of the story and in which way. Indeed, the actor was never alone in the room during the audio recording or the motion capture and was in fact telling the stories to one or two experimenters.

### Apparatus

The motions of the actors were recorded with the Xsens MVN suit [46] in a large, quiet room. Xsens MVN is a full body suit made of lycra, it includes 17 sensors aligned with anatomical landmarks of the body (see Figure 1). Each sensor is composed of an accelerometer, a gyrometer and a magnetometer. The placement of the sensors and corresponding cables in the suit enables the user to perform unrestricted actions. Two master units in the back of the suit transmit the data from the sensors wirelessly to a computer, where the software MVN Studio [46] maps the captured data onto an actor-scaled human skeleton-like 3D model and records the resulting motion output in real-time, at 120 FPS. Although inertial motion capture systems can introduce errors in the absolute position of the suit wearer in space, the data they provide for relative orientation, acceleration, and velocity are reliable. In our motion capture setup the actor was seated on a stool, thus the possible drift was of no consequence since the centre of the skeleton was stationary in real world.

The motion capture sessions were also video recorded with the permission of the actors. Due to the privacy protection of the actors, the video and audio recordings were used only for reference. Another important function of the the video camera and the microphone in the setup was to create an impression that every aspect of emotion expression, e.g., prosody and facial expressions, were important for the research purposes of the study. Using this immersive and multimodal setup we aimed to increase the probability of the actors expressing the emotions in a more natural way, without intentionally exaggerating or suppressing any of the emotion expression channels.

The open source presentation software used for acting scripts display (PsychoPy [56]) and the motion capture software (MVN Studio) ran on a Dell Precision M6400 laptop (Intel Core2 Duo 2.8 GHz, 4GB RAM, nVidia Quadro FX 3700M graphics card with 1024MB VRAM). The laptop was attached to a 20″ Dell external monitor with a resolution of 1280×1024 pixels which was used to show the current utterance or acting motivation and the corresponding emotion category label to the actors. An external keyboard and pedals, designed for the experiment, were connected to the laptop via USB (Figure 1).

### Motion Capture

Eight actors (4 female), 20 to 33 years old (M = 25.6, SD = 4.24) participated in the motion capture sessions. Informed written consent was obtained from each actor regarding their audio, video, text annotation, and motion capture data. Only the latter two types of data, due to privacy reasons, are available to the community. All actors received monetary compensation for their participation and none were aware of the end purpose of the motion capture until all the data was collected. After that the actors were offered debriefing concerning the goal of the study. All actors had normal or corrected to normal visual acuity, the male actors and one female actor originated from Germany, one female actor came from India, one - from England, and one from Ireland. All participants' command of English was either at the native speaker's level or high enough to enact the English texts fluently and without difficulties.

After having annotated the three chosen texts with the available emotion categories, each actor came for the first motion capture session, where they were given four types of short scenarios to act out. Each scenario type encompassed ten emotion categories (all the categories listed in the previous section with the exception of *neutral*). The emotion categories were randomised for each scenario and each actor. In each scenario instruction, the actor would see the goal emotion and the description of the situation, so the actors did not have to interpret and label the emotional content of the short scenarios themselves. The short scenarios largely resemble classical emotion induction vignettes used in related literature, e.g., [48]. These scenarios are later compared to the coherent narrations. The scenario types increase in their verbal content and subtlety of emotions and were performed in the following order:

1. Solitary **non-verbal** emotional scenarios (**NS**): the instructions indicated that the actor was to imagine that he or she was alone. Example: (*Pride*) “You are sitting alone in your room at your desk and have just finished doing an online IQ test. According to the results, you are among the 5% smartest people in the world!”
2. Communicative **non-verbal** emotional scenarios (**NC**): the instructions indicated that the actor was in company with a friend/friends/a colleague/colleagues. Example: (*Surprise*) “You are at a cafe catching up with a friend you haven't seen for a long time. She tells you that she is now doing skydiving and ice climbing.”
3. Short **sentences** without direct speech (**SN**): The actor was asked to act out a short preselected sentence from a fairy tale, the sentence contained no direct speech, the person acted on behalf of the narrator of the story. Example: (*Joy*) “One of Rapunzel's tears fell on prince's face and he could see again.”
4. Short **sentences** with direct speech (**SD**): The actor was asked to act out a short preselected sentence from a fairy tale, the sentence contained direct speech, the person acted on the behalf of a character and, if needed, the narrator of the story as well. Example: (*Fear*) “ “Please don't kill me!” – cried the rabbit – “I might be useful to you in the future!” ”

In the three motion capture sessions that followed each actor worked on one story at a time (see Table 3 for the distribution of stories among actors). The text of each story was shown phrase by phrase in its natural order as was determined by the previous delimitation and annotation processes (see section Texts). Each phrase was shown together with the emotion label that had been assigned to it by the actor during the fairy tale text annotation process, e.g., Joy*: “…you have a kind heart…”*.

**Table 3:** Stories narrated by actors during motion capture sessions.

Story Title	AnBh	DiMi	HeGa	LeSt	MaMa	NoVo	PaPi	SiGl	Count
Blue Beard (TBB)	√				√	√	√		4
Flower Princess (TFP)	√	√	√				√		4
Golden Goose (TGG)		√	√	√					3
Hoodie Crow (THC)				√	√				2
Jack My Hedgehog (TJH)						√	√	√	3
Owl And Eagle (TOE)					√				1
Six Swans (TSS)								√	1
Swineherd (TSH)			√					√	2
White Duck (TWD)	√	√		√		√			4
Sum	3	3	3	3	3	3	3	3	24

Each actor performed three stories in total, one story per session. To increase actors' motivation and comfort, the choice of three stories out of 9 was with the actor and not the researcher, which resulted in the fact that some stories were acted out by several actors (e.g., TBB, TWD) and some by only one actor (e.g., TSS).

In all recording sessions, each scenario type and story narration was rehearsed and recorded two to three times, in order to allow actors to get used to the text of the story and the emotion flow, but only the last recording was used later for the categorisation study and the motion capture dataset compilation. During the recording sessions the actors were seated on a stool 1.5 meters away from the Dell monitor (Figure 1). The size of the text on the screen was large enough for the actors to read it effortlessly. They progressed through short scenarios or coherent stories by pressing the right foot pedal. This allowed them to maintain their own performance speed and also kept their upper body free for the full range of emotion expressions. The timing of the pedal presses was recorded for the synchronisation of acting script presentation and the motion capture stream. Especially in narration tasks, in order to minimise the risk of asynchrony between the actor's progress through the story and the recorded pedal presses, we instructed the actors to finish acting out each phrase before proceeding to the next one. It is important that, although the actors felt involved in the narration process, they were not required to recall personal emotional memories — a technique often used for emotion induction, which could have affected actor's overall emotional state if some particularly strongly unpleasant emotional memories had been recalled.

### Motion Capture Files Format

In our database the motion sequences are available in the following formats:

- **BVH** (Biovision Hierarchy) was developed by a motion capture company called Biovision. BVH is one of the most popular motion data formats and is mainly used as a standard representation of movements in the animation of humanoid structures. A typical BVH file can be viewed in any text editor. Each file starts with a skeletal hierarchy, which begins with the “root” node and continues to nest child “joint” bones. The number of joints and structure of the hierarchy solely depend on the previous motion capture recording and/or export setup. “Offset” describes the offset of each joint from its parent. “Channels”, typically six for the root (position and orientation) and three for the rest of the joints (orientation only) describe the motion data. BVH rotations are recorded in Euler angles instead of quaternion terms. The keyword “motion” marks the beginning of the motion data. Number of frames and the frame time (sampling rate) are also given. Our files have 0.008333 frame rate, or 120 frames per second. The rest of the file contains the actual motion data. Each line is one sample of motion data in one frame. The numbers appear in the order of the channel specifications as the skeleton hierarchy was parsed. The hierarchy in our BVH files has 22 joints with 3 channels each and the root with 6 channels. Thus, every motion data line has 72 numbers.
- **MVNX** (Moven Open XML format) contains position, orientation, acceleration, velocity, angular rate, and angular acceleration of each joint (also called segment) in 3D. The files have XML structure with elements such as “<segment>” or “<frames>” and attributes to the elements, e.g., “<segment id = “1” label = “Pelvis”>”. The MVNX files consist of several major sections. The “mvnxInfo” section contains overview information on the number of frames, frame rate and other descriptive information. The “meshScale” section is used for visualisation of the character in Moven Studio only, while “segments” defines all positions of joints. Unlike the BVH format, the “segments” section is not hierarchical, instead, each segment has several points with their individual offset positions with respect to the origin of that segment. The “frames” section contains the actual motion data, where each frame is represented by one row containing 23 segments, 7 channels in each segment: 4 for quaternion orientation, 3 for the position. The last two values are the time of the frame in milliseconds and the timecode. Both time values rarely start at 0 in our data since motion sequences originate from longer motion caption sessions and the original timing values were retained. All values are set in the global coordinate system. Additionally, kinematic information is available in 1×3 vector form for each segment for velocity (*m*/*sec*), acceleration(*m*/*sec* ^2^), angular velocity (*rad*/*sec*) and angular acceleration (*rad*/*sec* ^2^) in corresponding file sections.

## Results

### Emotion Categorisation Study and Motion Sequences Selection

We obtained seven motion capture files from each actor — four sets of short scenarios and three coherent stories. The files were then split into smaller sequences according to pedal press timestamps. The short scenario motion sequences were trimmed to cut away the time stretches before and after the motion. In sum, from the short scenarios 320 motion sequences were obtained (10 emotions ×4 types of situations ×8 actors). The 24 coherent narrations (3 stories ×8 actors) were split into shorter motion sequences based on timestamps obtained from foot pedal presses. In total, 1380 motion sequences were selected from narration motion capture data. The resulting 1700 motion sequences were further used for the emotion categorisation experiment which was part of a larger perceptual study. While the results of the experiment are given in full detail in [51], here we will briefly report on its structure. We also provide details that were out of scope of the previous study but are highly relevant here.

**Figure 2:**
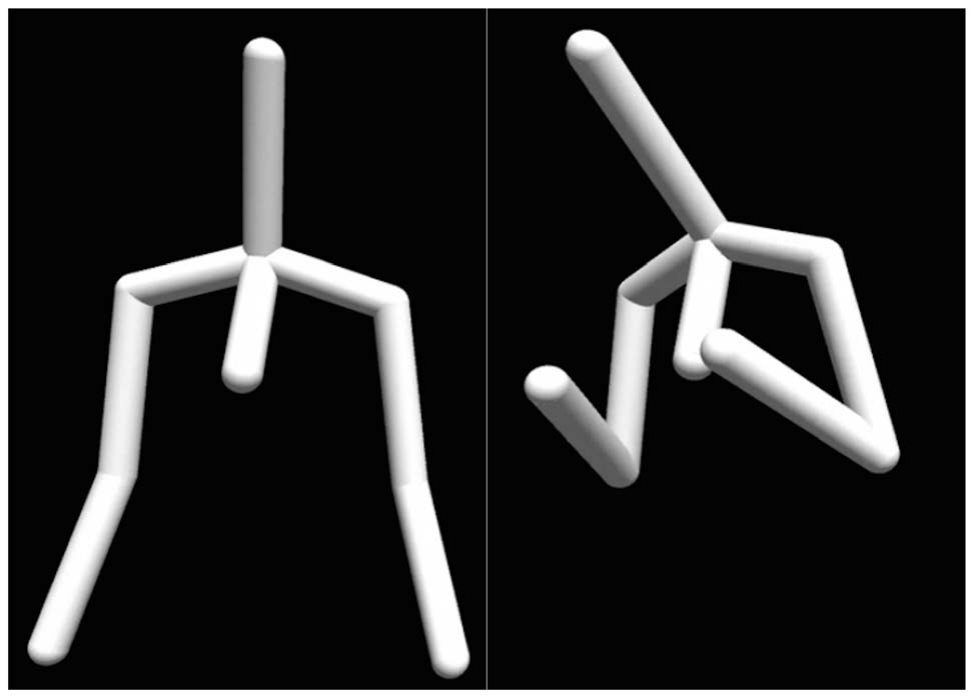
Stick-figure representations of human upper body used in the emotion categorisation studies [51]. Regardless of the body proportions of the actor, the motion trajectories were mapped onto a skeleton of average body size. Note that the motion capture files included in the database contain data for the full body and have the original actors' body size and proportions.

The emotion categorisation study used the full dataset of 1700 motion sequences. The motion sequences were displayed as dynamic stick-figure representations of human upper-body (Figure 2 and Video S1). Fifty five participants (28 female) took part and each participant categorised a unique combination of 340 randomly chosen motion sequences. The stimuli blocks were organised in such a way that by the end of the experiment each of the 1700 motion sequences had been categorised by eleven participants. In every trial the participant was to choose one of the eleven emotion categories in a two-step response procedure, by first deciding whether the presented motion was *emotional* or *neutral*, then, if they had chosen the former, categorising the motion with one of the ten remaining emotion categories. The response could always be changed until the participant was satisfied and proceeded to the next trial.

**Figure 3:**
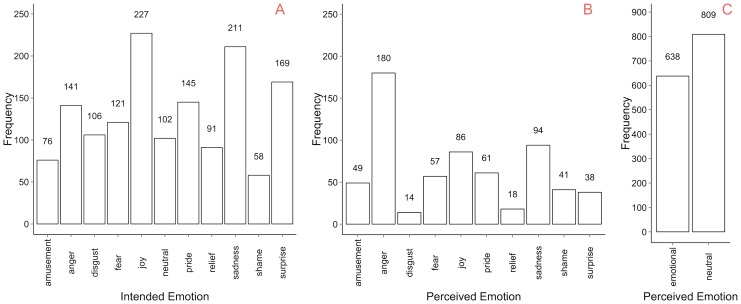
Motion sequence frequencies across intended (A) and perceived (B, C) emotions. Intended emotions originate from actors' text annotations while perceived emotions come from the categories forming a unique modal value in observers' response distribution for every motion sequence. The perceived emotion frequencies are split into two graphs to allow the same y-axis scale for (A) and (B) graphs. The *emotional* category in plot C is the sum of all frequencies in plot B.

As a result of the classification study, as many as 85% of motion sequences have a unique modal value in the distribution of observers' categorisation. The category of the unique modal value is henceforth referred to as *perceived emotion*, while the emotion category obtained from the actor's annotation corresponding to the sequence is referred to as *intended emotion*. The frequency of the intended emotion categories as indicated by the actors for every selected motion sequence during the annotation phase is shown in Figure 3A. The distribution of perceived emotion across emotion categories in the database is given in Figure 3B and Figure 3C.

The distribution of the perceived emotions across categories is very different from the distribution of intended emotions as indicated by the actors. The observed agreement between the intended and the perceived emotion categories is 19%. This result suggests that the emotion intended by the actor is often perceived as a different category by the observer. It further highlights the importance of recording the intended emotions in the context of situational emotion expressions that are not based on predefined or exaggerated emotions, along with the perceived emotions based on the same motion sequences. The current database provides this information.

In our final database we included those motion sequences that have a unique modal value in the observers' response distribution, the total number amounting to 1447 motion sequences. While 85% of motion sequences have a unique perceived emotion category, the number of observer responses falling into the modal category varies. We define the measure of *consistency* as the proportion of observers' responses that fall into the most frequently chosen emotion category. For example, if for a given motion sequence six out of eleven participants categorised the sequence with the same category, consistency equals 6/11 = 0.545. Note that, in cases when the response distribution is bimodal or multimodal (i.e. more than one category received the same number of responses), consistency is not defined.

**Figure 4:**
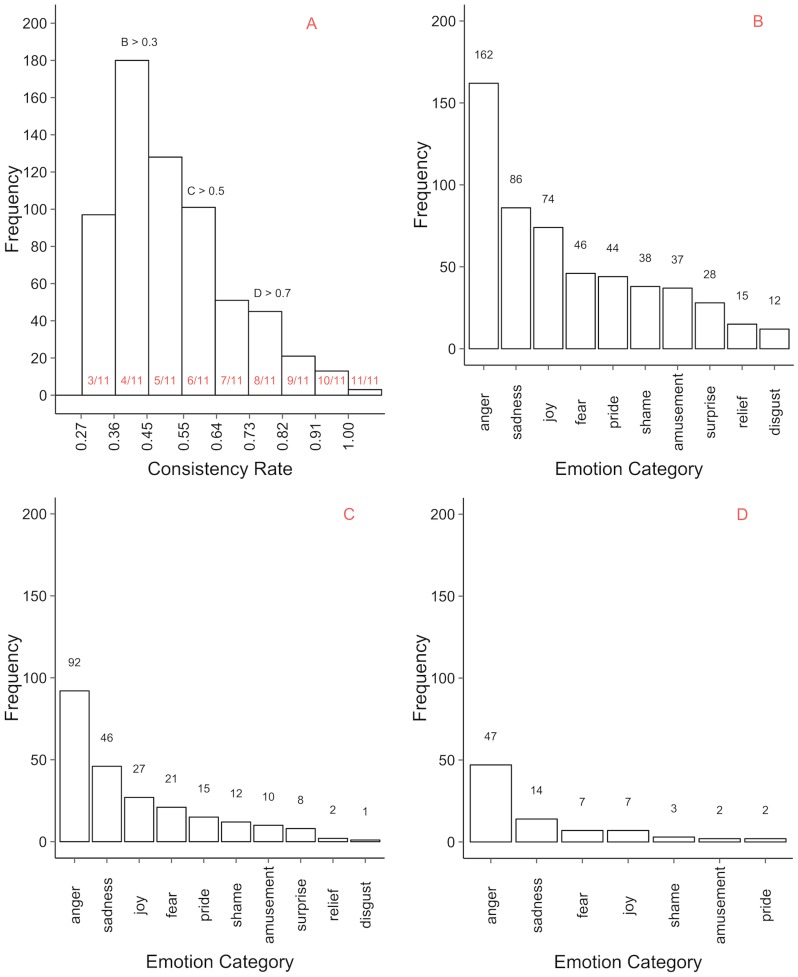
Emotion frequency distribution across consistency levels. (A) Histogram of consistency rates across motion sequences. The minimally possible consistency is always equal to one divided by the number of observations for the given stimulus and multiplied by two because there have to be at least least two observers assigning the same category to the stimuli to form a modal value. (B) Distribution of perceived emotions across categories with a consistency rate of 0.3 or more. (C) Distribution of perceived emotions across categories with a consistency rate of 0.5 or more. (D) Distribution of perceived emotions across categories with a consistency rate of 0.7 or more.

The consistency is useful if the researcher wants to investigate the perception of emotions from body motion, for instance, to identify motion sequences or emotion categories that are perceived similarly by different observers. Our database organisation makes it easy to extract motion sequences with user-selected perceived categories and/or motion sequences with defined consistency rates. Figure 4 (A) shows the distribution of consistency rates across motion sequences perceived as *non-neutral*, showing that 0.3 is the most frequent consistency rate. Applying three cut-off levels (0.3, 0.5 and 0.7) we show the frequency of ten *non-neutral* emotion categories that include motion sequences with the cut-off consistency rate or higher (Figure 4 B, C, D). Motion sequences included in Figure 4C are a subset of motion sequences from Figure 4B and motion sequences from Figure 4D are a subset of motion sequences from Figure 4B and Figure 4C. *Anger* and *sadness* are always the most frequent perceived emotion categories, indicating that the observes of the corresponding motion sequences were in high agreement with each other. On the contrary, *surprise, relief* and *disgust* are the least frequent and are not even present in Figure 4D, meaning that though these categories formed the modal value in the observers' responses for certain motion sequences, the latter were not numerous and the number of responses falling into the categories was never more than half of the total responses available.

The perceptual study that focused on the categorisation of emotion categories, its results and implications are described in detail in [51]. In the rest of this section we analyse two fundamental sources of variability: the acting tasks they originated from and the actors they were performed by. For each of those sources we provide the following data: observed agreement between perceived and indented emotions (referred to as *recognition accuracy*), consistency rates, and physical motion properties. The latter are represented by four features: duration (in seconds), peaks (mean number of peaks and valleys across the *x*, *y*, *z* trajectories for each joint in question), speed (m/sec), and span (average distance between the joints, m). For the purposes of simplicity, here we used only the left and the right wrist joints of the underlying joint configuration for the motion properties extraction, as these were the most active joins in the motion production and are thus most suitable for motion characterisation.

### Motion Sequences Distribution

Tables 4–6 display the distribution of the motion sequences included into the new database across actors, intended emotions and acting tasks. Table 4 shows frequency of motion sequences across emotion categories and acting tasks, while Table 5 shows each actor's final contribution to the database across acting tasks and specific stories. The column name abbreviations stand for acting tasks described earlier in section *Motion Capture* and in Table 3. Table 6 shows how many motion sequences were contributed to the final dataset by each actor across the emotion categories they intended to express. The next section gives detailed information on the results of the emotion categorisation study, focussing on perceived emotion categories across acting tasks and individual actors.

**Table 4:** Frequencies in the final set of motion sequences across intended emotion categories and acting tasks.

Emotion	Short scenarios				Narrations								
	Non-verbal		Sentences										
	NS	NC	SD	SN	TBB	TFP	TGG	THC	TJH	TOE	TSS	TSH	TWD
amusement	7	6	8	6	4	31			8				6
anger	5	8	8	8	24	9	17	4	17		5	13	23
disgust	4	6	5	6	8	9	18	3	12	1		11	23
fear	5	7	7	8	29	12	4	4	6	2	6	3	28
joy	7	5	8	8	13	52	29	16	15	23	3	21	27
neutral					6	25	11	5	10	2		5	38
pride	8	5	8	8	18	39	13	1	17	6	2	12	8
relief	6	7	4	4	9	18	4	10	6	3	3	1	16
sadness	7	6	8	7	19	14	34	12	12	3	9	6	74
shame	6	7	7	8	3	8	8	2	4			2	3
surprise	7	6	5	7	20	18	26	21	12	4		11	32

The abbreviations stand for: NS — non-verbal solitary, NC — non-verbal communicative, SD — sentences with direct speech, SN — sentences without direct speech, TBB — Blue Beard, TFP — Flower Princess, TGG — Golden Goose, THC — Hoodie Crow, TJH — Jack My Hedgehog, TOE — Owl and Eagle, TSS — Six Swans, TSH — Swineherd, TWD — White Duck.

**Table 5:** Frequencies in the final set of motion sequences across actors and acting tasks.

Actor	Short scenarios				Narrations								
	Non-verbal		Sentences										
	NS	NC	SD	SN	TBB	TFP	TGG	THC	TJH	TOE	TSS	TSH	TWD
AnBh	7	8	9	10	98	67							93
DiMi	9	9	9	9		57	48						52
HeGa	9	7	9	8		57	35					54	
LeSt	7	9	8	8			81	78					84
MaMa	9	7	8	9						44			
NoVo	8	8	8	8	55				41				49
PaPi	6	7	7	10		54			47				
SiGl	7	8	10	8					31		28	31	

The abbreviations stand for: NS — non-verbal solitary, NC — non-verbal communicative, SD — sentences with direct speech, SN — sentences without direct speech, TBB — Blue Beard, TFP — Flower Princess, TGG — Golden Goose, THC — Hoodie Crow, TJH — Jack My Hedgehog, TOE — Owl and Eagle, TSS — Six Swans, TSH — Swineherd, TWD — White Duck.

**Table 6:** Frequencies in the final set of motion sequences across actors (rows) and intended emotion categories (columns).

	AnBh	DiMi	HeGa	LeSt	MaMa	NoVo	PaPi	SiGl	total
amusement	7	3	4	4	4	16	35	3	76
anger	30	22	20	15	4	24	6	20	141
disgust	14	11	13	30	3	8	12	15	106
fear	40	14	9	17	5	16	8	12	121
joy	52	27	33	46	26	10	17	16	227
neutral	25	10	18	20	2	12	10	5	102
pride	27	21	23	10	10	19	23	12	145
relief	25	14	4	22	6	5	6	9	91
sadness	41	31	15	61	6	30	8	19	211
shame	7	13	5	13	4	3	5	8	58
surprise	24	27	35	37	7	34	1	4	169
total	292	193	179	275	77	177	131	123	1447

### Emotion Recognition and Consistency across Acting Tasks and Actors

**Figure 5:**
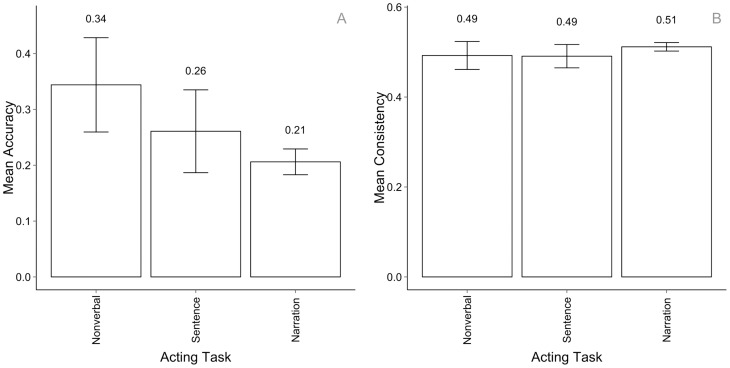
Emotion recognition accuracy across acting tasks for intended emotions (A) and consistency rates for perceived emotions (B) across acting tasks. All error bars represent 95% CI.

In this section we organise motion sequences into three groups according to the tasks they were produced in: non-verbal, short sentences and narrations (see also Table 4). Figure 5 shows that non-verbal motion sequences have received higher recognition accuracy (0.35, with 1.0 being the absolute possible maximum) than their counterparts that included speech (0.25 and 0.21 for short sentences and narrations respectively). This suggests that it was easier for the observers to recognise the actor's intended emotion when it was expressed non-verbally. The consistency however is on average the same across tasks: 0.49, 0.49 and 0.51 respectively (Figure 5), showing that observers had relatively high agreement rates among each other for all motion sequences even when they disagreed with the actors.

**Figure 6:**
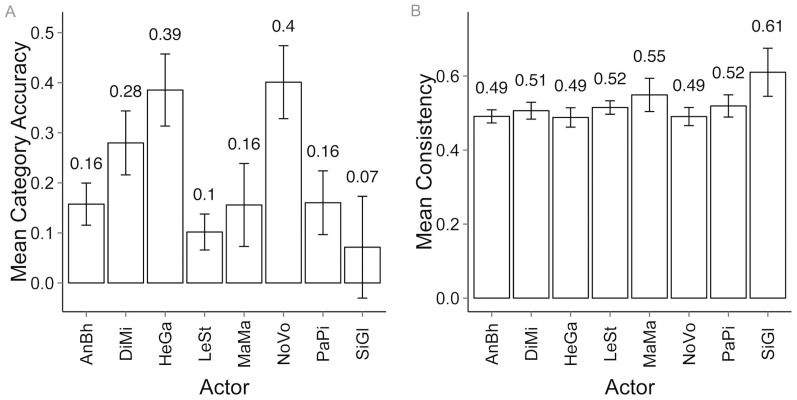
Average recognition accuracy across actors (A) and observers' response consistency across actors (B). All error bars represent 95% CI.

The actors who took part in this research were young adults with varying acting experience (from “currently active amateur” to “amateur in the recent past”). As one can see, recognition accuracy rates vary greatly among individual actors, ranging from 0.07 to 0.4 (also see Table 7), but the consistency is comparable across all actors (Figure 6). Nevertheless, in the final database, the number of motion sequences coming from each actor ranges from almost three hundred (292 from “AnBh”) to under one hundred (77 from “MaMa”).

**Table 7:** Frequencies of motion sequences across actors where intended and perceived emotion categories coincide, sorted by the total frequency within each emotion category (rows) and actor (columns).

	NoVo	HeGa	DiMi	AnBh	LeSt	SiGl	PaPi	MaMa	total
neutral	9	18	9	18	20	3	9	2	88 (.86)
anger	12	17	9	9	5	7	2	4	65 (.46)
sadness	15	1	16	8	1	2	2	2	47 (.22)
fear	14	3	10	3		2			32 (.26)
joy	7	14	2	2	1	2		1	29 (.12)
pride	4	10	1	3	1	1	3	1	24 (.16)
surprise	9	3	1					2	15 (.08)
amusement		2		2		1	3		8 (.10)
shame			5	1		1	1		8 (.14)
disgust	1		1			1	1		4 (.03)
relief		1				2			3 (.03)
total	71 (.40)	69 (.38)	54 (.28)	46 (.15)	28 (.10)	22 (.16)	21 (.16)	12 (.15)	323 (.22)

Values in round brackets represent the proportions of the frequencies in relation to the whole database (see Table 6).

### Physical Properties of Motion Sequences across Acting Tasks and Actors

**Figure 7:**
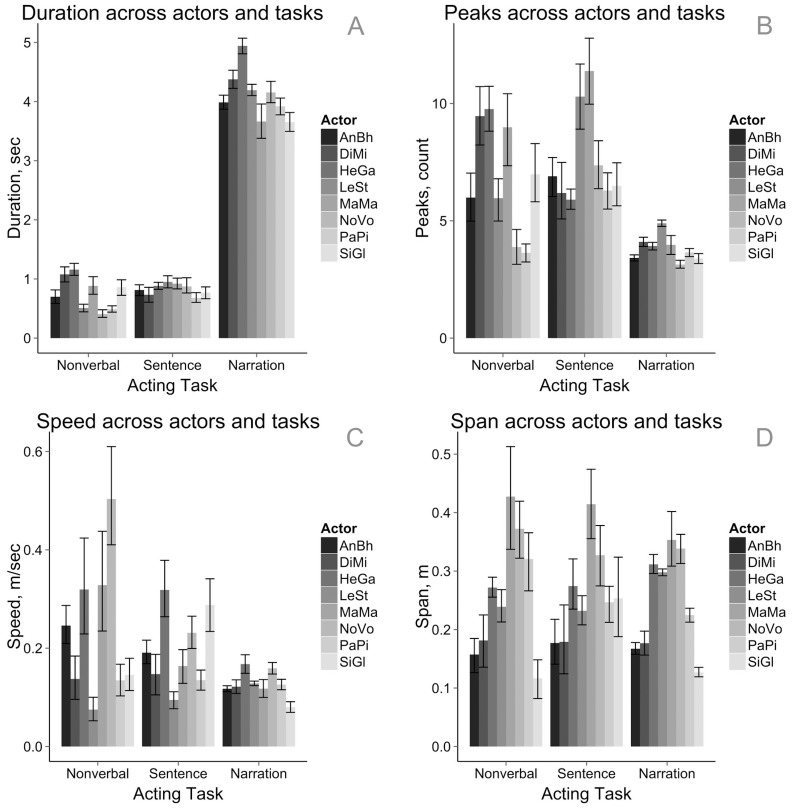
Physical properties of motion sequences across acting tasks and individual actors. All error bars represent 95% CI. The panels show: (A) Duration, sec; (B) Peaks in motion trajectories of right and left wrists across acting tasks; (C) Average motion speed; (D) Average motion span. As the bar plots show, physical properties of motion sequences depend both on the acting tasks and on the individual actors.

Figure 7 shows motion properties across the three acting tasks. On average, motion sequences have longer duration in the narration task than in short scenarios, and also lower speed and number of trajectory peaks. Such slower and smoother profile in narration scenarios can be probably explained by the fact that the motion produced during narration was accompanied by coherent speech. Additionally, the narration task is the only of the three acting tasks that contained *neutral* motion as indicated by actors' emotion annotations. Note however, that there is almost no difference in motion span across the acting tasks, showing that each individual actor used approximately the same space volume during all the motion capture sessions regardless of the acting task.

Actors vary not only across recognition rate by the observers, but also by their ways to produce body motion, which is reflected in Figure 7. While the duration of motion sequence is mostly uniform among the actors (M = 3.42, SD = 0.55, min  = 2.47, max  = 4.21), motion speed, peaks in motion trajectories and especially motion span vary greatly among individual actors (speed, *m*/*sec*: M = 0.14, SD = 0.03, min  = 1.18, max  = 1.96; span, *m*: M = 0.26, SD = 0.09, min  = 0.14, max  = 0.38; number of peaks, *count*: M = 4.62, SD = 0.99, min  = 3.58, max  = 6.65). The variation in span among actors can be partially due to the differences in individual motion style, but also due to different body configurations, e.g., arm span. The relatively uniform duration of motion sequences can be explained by the fact that the actors performed the same short scenarios and also did not vary greatly in the utterance length in narration tasks.

### Final Database Format

All motion sequences are available to the community, each motion sequence coming with the additional meta-information that describes its various aspects, e.g., the physical properties of the motion, the intended emotion according to the actor and the response categories from the participants of the categorisation study. The information about the source of the motion sequence is also presented — the acting task the sequence comes from (short scenario or a full narration with the story title) and for most motion sequences what exactly the task was, namely, the motivation sentence for short scenarios and the specific phrase that was acted out during the production of that motion sequence. Some information about the actors is available as well, such as the actors' code names, their gender, age, and mother tongue. Table 8 gives an overview of available information for each motion sequence. The user can filter through and search for the information in the fields and select a subset of the motion sequences. For each selected motion sequence various formats as well as the descriptive meta-data information are available for download (http://ebmdb.tuebingen.mpg.de).

**Table 8:** Online Database Overview.

Column Name	Description
Actor-dependent Motion Properties	
Motion Id	Unique motion sequence file name
Intended Emotion	One of the eleven emotion categories as intended by the actor
Intended Polarity	*Positive, negative* or *neutral* polarity of the motion sequence as intended by the actor
Duration	Duration of the motion in seconds
Peaks	Number of peaks and valleys in motion trajectory along *x*, *y*, *z* axes for the left and the right wrist joints
Speed	Average speed in m/sec for the left and the right wrist joints
Span	Average span in meters between the left and the right wrist joints
Acting Task	Nonverbal, Sentences, Narration
Acting Sub-task	Specific sub-task or story title (see Table 4)
Actor	The id name for one for the eight actors who performed the motion sequence
Gender	Actor's gender (“f” – female, “m” – male)
Age	Actor's age (*min* = 22, *max* = 30)
Handedness	Actor's handedness (“r” – righthanded, “l” – lefthanded
Native Tongue	Actor's mother language (German, English, Hindi)
Observer-dependent Motion Properties	
Perceived Category	One of the eleven emotion categories as perceived by majority of the observers
Perceived Polarity	*Positive, negative* or *neutral* polarity of the motion sequence as perceived by majority of the observers
Accurate Category	“1” when intended and perceived emotions coincide, “0” otherwise
Accurate Polarity	“1” when intended and perceived polarity coincide, “0” otherwise
Responses	The list of eleven responses to the motion sequence from all the observers
Consistency	The proportion of responses taken by the unique modal value, which is also recorded in “Perceived Category”
Text	The text that served as acting motivation (not spoken out loud in non-verbal tasks)

The 1447 motion sequences can be filtered and sorted by their metadata represented in the columns, such as intended emotion, perceived emotion, physical properties of the motion, actor information, etc. The database is available online (ebmdb.tuebingen.mpg.de), accompanied by usage instructions and the license agreement.

The database can be extended by adding new motion sequences and/or new properties of existing stimuli, such as ratings or categorisation labels from new experiments. We plan to add affect ratings to some of the stimulus material in near future. We welcome other researchers to contact us for new additions (either new recordings or newly measured properties of existing motion sequences). Our database is set up in such a way that little manual maintenance is required.

## Discussion

Our new database gives researchers access to human body motion patterns produced during emotional narrations. This database is unique and valuable for several reasons. First, the narration performance was kept as close to natural as possible. The actors were not aware that only their body motion was of primary importance to the authors. Their facial expressions and voice were recorded along with the body motion and the actors were narrating an unabridged story. Second, the rich set of emotion categories is another valuable feature of the database - we used not only the six basic emotions (*anger, disgust, fear, joy, sadness, surprise*) but expanded the list with four extra emotion categories: *amusement, pride, relief and shame* and added the category of *neutral*. Finally, the motion capture format itself is a big advantage for motion pattern analysis, as it depicts human motion in 3D space and at a high time resolution.

Researchers from several branches of science may find our database especially useful, i.e. psychologists, computer scientists, neuroscientists, and linguists. The motion sequences could be used in emotion perception research, motion analysis and synthesis, in behavioural and brain imaging studies. The motion capture took place in a naturalistic setting, ensuring that the resulting motion sequences represent expression of emotions via body language without unnecessary exaggeration. Thus, these patterns have a potential for model building in order to synthesise expressive natural looking motion for virtual character animation. Unlike video recordings, the motion capture format in our database allows one to display biological motion and change many of its properties, e.g. speed of selected trajectories, magnitude, scale and position in space relative to the rest of the body, and visual representation (point lights, full skeleton or parts of skeleton). Individual properties of the actors, e.g., their age, gender, body shape and other factors potentially confounding the results when pure human motion is in question. In motion capture format these properties are not available to the observer, but, could be added in a controlled experiment using animation tools. Since every motion sequence comes with the information about the actor who performed it (gender, age, native tongue), our data could be used for studying individual motion styles as well as differences and similarities of emotion expression across genders and cultures. The 24 annotations of texts can be used as a separate corpus for research in sentiment analysis or used together with the motion capture files to study human gesticulation when it accompanies meaningful speech, including the semantic aspect of gesture production.

However, our database is not without limitations. First, we cannot provide precise synchronisation between the text and the motion trajectories for each motion sequence that were produces in the *sentences* and *narration* acting tasks. The original motion capture sequences were split into shorter sequences based on the timing of the foot pedal presses. The pedal also allowed the actor to navigate to the next non-verbal motivation scenario, short sentence, or phrase of a story. Thus, the synchronisation between the text and the motion is only available for the phrase boundaries (see Motion Capture section in Materials and Methods for more detail). Video and audio recording are not available due to the protection of the actors' privacy. In the future, databases incorporating facial motion capture, fundamental voice frequency, the text being pronounced, and the body motion capture could allow researchers to study multimodal emotion expression in naturalistic narrative scenarios. The setting in which the motion capture took place is important to keep in mind for potential studies, since it is yet unknown in how far the narrative scenarios generalise to other situations of emotional body expressions, such as emotional gait patterns, expressive dancing, and social interaction in dialogues.

Our database provides not only the motion sequences labeled for actors' intended emotion displays and the original annotations of full narration texts, but also the perceived emotions obtained during an emotion categorisation study [51]. The participants of our categorisation study observed motion patterns of upper body of the actors mapped onto a stick-figure display of standardised size. Additionally, the observers did not watch the full narrations but were presented with short motion sequences in randomised order. The simplification of the stimuli was important as we aimed to investigate the amount of information the body motion itself brings into the emotion expression in narrative scenarios. As we found out, the participants were able to recognise the emotions at the above chance level even in the impoverished stimuli. The resulting categorisation responses from the observers are included in the database and are available as metadata with each motion sequence file. As each motion sequence was categorised by eleven observers, the most frequently chosen emotion category (the perceived emotion) is included in the database as a value separate from the full list of responses. The proportion of responses the perceived category (consistency) takes and its correspondence to the emotion intended by the actor (accuracy) are provided in the database as well.

## Conclusions

In this study we collected a large database of motion sequences from multiple actors by designing a way to elicit and record emotional body expressions in narrative scenarios. Related research [40], [48], [57] gained insight into how emotional body expressions are produced and perceived, but the used datasets of emotional portrayals were, sometimes deliberately, exaggerated and thus unlikely to occur in typical day-to-day experience. We aimed to collect natural emotional body expressions to deepen the understanding of the role of emotional body expressions in natural human communication. We thus chose coherent stories as the textual material for our study. The resulting emotional monologues are a close approximation to a bedtime story or an anecdote told to friends at a party, and not an attempt to reproduce stage acting settings.

We used eleven emotion categories, a richer set than in most related research, in order to allow actors to encode and express their perception of story texts in full detail. During the motion capture sessions the actors were free to express emotions through their speech, face and body. This freedom of emotion expression was an important aspect of our research, since we were interested in keeping the amount of information expressed via body motion at natural level of a typical narration scenario. Our database, available to the community, includes the motion capture files, the intended emotions expressed by the actor, and the perceived emotions determined by an emotion categorisation study.

## Supporting Information

Video S1 **Examples of motion sequences mapped on stick-figure representations of human upper body.** The motion capture files included in the database contain data for the full body and have the original actors' body size and proportions. The upper-body representations were used in the emotion categorisation studies [51], where the motion trajectories were mapped onto a skeleton of average body size and where independent of the body proportions of the actors.
(MP4)
